# Immunomodulation: precision targeting for restoring immune homeostasis and therapeutic applications

**DOI:** 10.1042/EBC20253042

**Published:** 2025-09-08

**Authors:** Fang Xu, Chao Wang

**Affiliations:** Institute of Functional Nano & Soft Materials (FUNSOM), Soochow University, Suzhou, 215123, China

**Keywords:** Immunomodulation, engineering, biomaterials, immunotherapy, nanotechnology

## Abstract

The intricate regulation of the immune system, maintaining equilibrium between pathogen defense and self-tolerance, is fundamental to health. Disruptions in this delicate balance underlie a vast spectrum of human diseases, extending beyond oncology to encompass autoimmune disorders, chronic inflammatory conditions, infectious diseases, allergies, and hypertension. While traditional therapies often rely on broad immunosuppression or direct pathogen eradication, the rapidly evolving field of immunomodulation offers a nuanced alternative: precisely calibrating immune responses to restore homeostasis or achieve targeted defense. This special issue comprises 12 review articles contributed by 57 international researchers, synthesizing key advances and emerging strategies for harnessing immunomodulation across diverse therapeutic applications.

Immune regulatory mechanisms play a central role in the pathogenesis, progression, and treatment of disease. Under homeostatic conditions, the immune system effectively eliminates pathogens and aberrant cells while preventing excessive reactions that damage host tissues. Dysregulation of this balance contributes to disease pathogenesis. For instance, Xu et al. systematically elucidate the significant contribution of autoimmune responses to hypertension and subsequent end-organ damage, such as an imbalance between regulatory T cells (Tregs) and effector T lymphocytes, the increased M1/M2 macrophage ratio, and heightened B-cell activity [1]. Current therapeutic development focuses on strategies targeting immune molecular regulation, including anti-inflammatory agents and vaccines, to mitigate hypertension progression and vascular injury.

Immunomodulation transcends the simplistic paradigms of global immune activation or suppression. It aims for precise intervention by targeting specific cell types, signaling pathways, or inflammatory mediators implicated in the disease process. This pursuit of specificity promises enhanced therapeutic efficacy while minimizing the debilitating side effects associated with non-selective immunosuppressants or cytotoxic therapies. Consequently, a diverse array of cutting-edge immune regulation strategies has emerged. These strategies increasingly utilize engineering approaches to deliver regulatory signals with high precision to target cells (Mckee et al*.*) [2], tissues (Zhao et al*.*) [3], or pathways (Yue et al*.*) [4], moving beyond systemic wide-net effects. Technologies like single-cell sequencing (scRNA-seq) are proving crucial in immunotherapy, particularly for efficacy prediction and biomarker discovery, as well as for assessing the impact of biomaterials on the immune microenvironment (Zhou et al*.*) [5]. This approach has catalyzed the development of a new generation of sophisticated immunomodulatory strategies designed to intervene with pinpoint accuracy.

Enhancing the activity and persistence of specific effector cells (e.g. tumor-infiltrating lymphocytes and pathogen-specific T cells) remains a critical therapeutic objective. Persistent challenges, especially within solid tissues and chronic disease settings, drive innovations in cell engineering, advanced delivery methods (e.g. biomaterial scaffolds), and safety controls (e.g. suicide genes) to broaden applicability. Mckee et al. elaborate on optogenetic strategies that enable dynamic control over T cell activation, cytokine production, and cytotoxic responses through targeted modulation of T cell receptor signaling, ion channels, transcriptional programming, and antigen recognition, thereby improving cancer immunotherapy efficacy while minimizing adverse effects [2].

Nanoplatforms offer sophisticated solutions for targeted immunomodulation. Advances in nanotechnology and drug delivery, including lipid-based nanoparticles (LNPs), polymeric micelles, and exosome-based carriers, facilitate efficient delivery of immunomodulators like Stimulator of interferon genes (STING) agonists, inducing potent anti-tumor immunity (Yue et al*.*) [4]. Nanocarriers can overcome barriers in challenging microenvironments, such as the dense extracellular matrix, immunosuppressive cells, and hypoxia in pancreatic ductal adenocarcinoma, by enhancing drug penetration, modulating immune cell function, and reprogramming the tumor microenvironment (Lin et al*.*) [6]. Engineering nanoparticles for specific delivery of immunomodulatory payloads (drugs, nucleic acids, and antigens) to immune cells within lymphoid organs or inflamed tissues enhances potency and reduces off-target effects. LNPs, as the leading mRNA vaccine delivery system, continue to evolve through lipid design optimization, formulation refinement, and selective organ-targeting strategies, improving safety, transfection efficiency, and biodistribution (Li et al*.*) [7]. Zhao et al. highlight how nanoparticle physicochemical properties (size, charge, hydrophilicity, and deformability) and surface modifications critically influence lymph node targeting efficacy. Surface engineering, employing biological (e.g. ligands) and chemical strategies, leverages receptor–ligand interactions to significantly enhance targeting precision and therapeutic outcomes [3]. Furthermore, the immunomodulatory roles of metal ions in regulating cancer cell death and stimulating immune cells present opportunities for novel metal ion-based therapies. Metal co-ordination polymer nanoparticles provide a promising platform for reformulating metal ions into drug-like entities, improving their *in vivo* pharmacological performance and therapeutic index in cancer therapy (Zhang et al*.*) [8].

Natural nanoparticles, particularly extracellular vesicles (EVs) derived from immune cells (iEVs), play crucial roles in immunoregulation. Wang et al. provide a comprehensive overview of the functions of iEVs from diverse immune cell origins and underlying mechanisms. iEVs contribute to immune activation by driving immune cell development and activation, enhancing antigen presentation via direct and cross-dressing mechanisms, and acting as signaling entities within immunological synapses to amplify immune responses [9]. Conversely, iEVs participate in immune regulation by modulating immune checkpoint molecule expression and transporting immunosuppressive cytokines and microRNAs to maintain homeostasis and mitigate excessive inflammation. Shah et al. summarized various immunoregulatory cell-derived EVs for autoimmune diseases therapy, such as Tregs, dendritic cells, mesenchymal stem cells, and neutrophils [10]. In addition to naturally occurring EVs, artificially modified EVs can enable more precise and efficient immune modulation, for example, by genetically engineering parent cells and isolating derived EVs. A major limitation is the batch-to-batch variability and poor yield. To overcome these challenges, researchers are actively developing more efficient EV collection and purification approaches, or improving the precise targeting of EVs to specific tissues or cells, ensuring efficient delivery while minimizing off-targeting side effects and reducing the required therapeutic dose.

In addition, the application of microorganisms in disease treatment is also a crucial aspect that deserves exploration. Engineering strategies are enhancing the ability of probiotics to target inflammatory sites. Approaches include surface physicochemical and biological coatings, genetic engineering, and the use of outer membrane vesicles (OMVs), minicells, and bacterial ghosts. These enable precise delivery or *in situ* synthesis of therapeutic molecules, expanding the multifunctional potential of probiotics (Wu et al*.*) [11]. Bacterial OMVs, naturally released lipid bilayer nanoparticles, exhibit strong immunogenicity, effective delivery capacity, and versatile engineering capabilities, making them attractive tumor vaccine platforms. While significant progress has been made in engineering OMVs to enhance tumor antigen presentation and therapeutic efficacy, challenges remain regarding reactogenicity, antigen expression optimization, and addressing tumor heterogeneity (Yang et al*.*) [12].

In conclusion, immunomodulation represents a paradigm shift in treating diseases rooted in immune dysfunction. By advancing beyond broad suppression toward the precise calibration of immune responses, this field offers the potential for more effective, durable, and safer therapies across a wide spectrum of conditions. The convergence of immunology, bioengineering, nanotechnology, microbiology, and data science is driving rapid innovation. Sustained research and translational efforts focused on overcoming current challenges are essential to fully realize the transformative potential of immunomodulation in reshaping the therapeutic landscape for patients suffering from immune-mediated diseases.

## Open Access

This article has been published open access under our Subscribe to Open programme, made possible through the support of our subscribing institutions, learn more here: https://portlandpress.com/pages/open_access_options_and_prices#conditional
